# Increased uptake on ^99m^Tc bone scintigraphy in a case of tumoral calcinosis in a child

**DOI:** 10.1259/bjrcr.20150012

**Published:** 2015-06-29

**Authors:** N Jawad, M Dumba, P Brock, K McHugh

**Affiliations:** ^1^Department of Radiology, Great Ormond Street Hospital for Children NHS Foundation Trust, London, UK; ^2^Department of Oncology, Great Ormond Street Hospital for Children NHS Foundation Trust, London, UK

## Abstract

Tumoral calcinosis is an idiopathic condition resulting in the periarticular deposition of calcium crystals and salts in soft tissues. It is rare in children, and even rarer in idiopathic form. We present a case of a 2-year-old female with tumoral calcinosis in the supraclavicular region, and, in particular, focus on the pertinent radiological findings with radiography, MRI and bone scintigraphy.

## Clinical presentation

A Kuwaiti female child, residing and receiving treatment in Kuwait, initially presented aged 20 months with a 6-month history of an enlarging left supraclavicular mass. There was no associated trauma or preceding illness. She was a term baby with an uncomplicated birth. All vaccinations were up to date. There was no family history of malignancy. On examination, she was systemically well. A 5-cm painless, firm mass was palpable in the left supraclavicular fossa. It was fixed to the underlying tissue, but not the overlying skin. There was no overlying erythema. Biochemical testing of the blood showed mildly raised Ca at 2.52 mol l^-1^ (normal range 2.17–2.44 mol l^-1^) and erythrocyte sedimentation rate at 34 mm h^-1^ (1–10 mm h^-1^) but the phosphate level was normal. Remaining full blood count and renal function tests were normal.

## Differential diagnosis

The differential diagnosis suspected in this case was a neoplastic process originating from bone or soft tissue.

## Imaging findings

**Figure 1.**
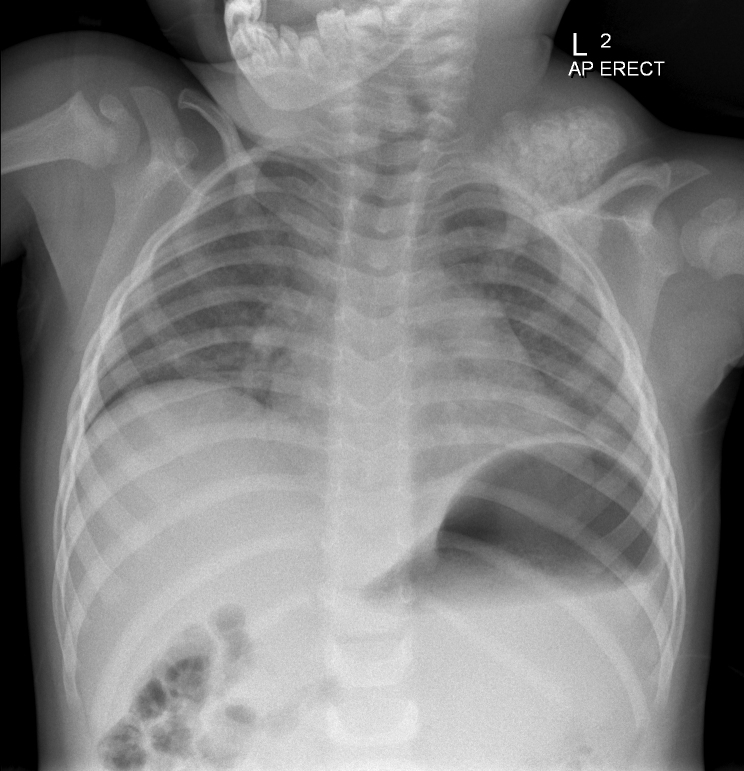
Admission chest X-ray showing a large soft-tissue mass containing heterogeneous calcification in the region of the left shoulder joint.

Initial neck ultrasound demonstrated a large, complex, densely calcified left supraclavicular mass. Subsequent plain radiography showed a 4-cm lobulated, heterogeneously calcified mass (Figure 1).

**Figure 2.**
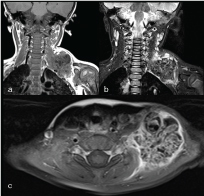
Coronal *T*_1_ (a), coronal short tau inversion-recovery (b) and axial *T*_1_ post-gadolinium MRI (c) showing a left supraclavicular mass with multiple low-signal areas consistent with calcification.

**Figure 3.**
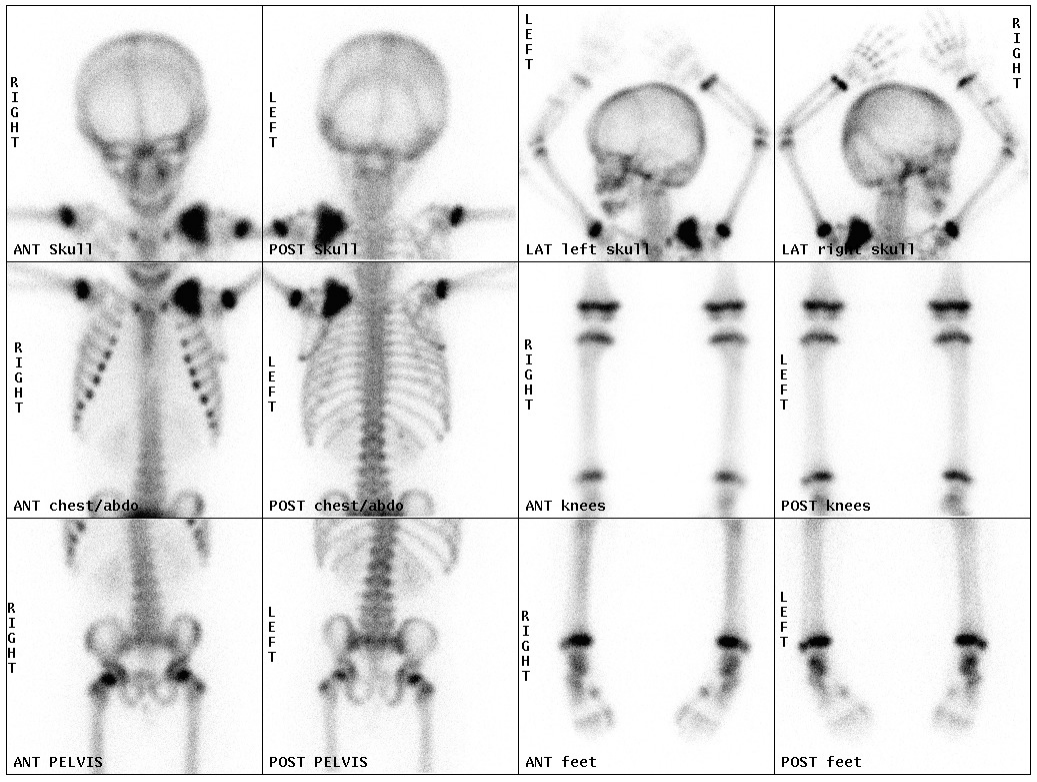
Bone scintigraphy (with ^99m^Tc) showing avid uptake in the left supraclavicular mass with normal uptake in the remaining bony skeleton.

MRI showed a 4.6 x 4 cm low *T*_1_, *T*_2_ and short tau inversion-recovery signal mass in the left retroclavicular region (Figure 2). On diffusion sequences, there was minimal restriction, which suggested a benign rather than malignant process. On nuclear medicine metaiodobenzylguanidine (MIBG) iodine-123 single photon emission computed tomography-CT imaging, there was no MIBG uptake. Although the mass was in close proximity to the brachial plexus, no bony destruction or invasion was demonstrated. Bone scintigraphy with technetium-99m (^99m^Tc) revealed avid uptake in the mass, with normal uptake in the remaining skeleton (Figure 3).

## Treatment

An excision biopsy was arranged for definitive diagnosis. Histology showed extensive necrosis, dystrophic calcification and confirmed tumoral calcinosis. No tumour was identified.

## Outcome and follow-up

The excision was curative and a recent CT study has confirmed the mass has not recurred.

## Discussion

The term tumoral calcinosis was first coined by Inclan et al in 1943.^1^ The pathogenesis of the condition is the deposition of calcium crystals and salts in the soft tissues around large joints.^2^ The most commonly affected large joints are the hips, pelvis, elbows and shoulders. Spinal involvement is rare. The condition is found more commonly in those of Afro-Caribbean origin.^1^ The idiopathic form of the condition is rare, and even rarer in children.

There are three main forms of the condition described by Smack et al.^3^

1. Primary normophosphataemic tumoral calcinosis. As in our case, these cases are sporadic with normal biochemical testing.
2. Primary hyperphosphataemic tumoral calcinosis.J These cases are likely to be familial (autosomal Jdominant or recessive), and are seen more in JAfro-Caribbeans and males. Biochemistry Jreveals an elevated serum phosphorus, normal Jserum calcium and normal parathyroid Jhormone assay. Up to one-third of cases are thoughtJ to be familial.
3. Secondary tumoral calcinosis. These cases are secondary to conditions such as chronic renal failure with secondary hyperparathyroidism, hypervitaminosis D, milk-alkali syndrome and bone destruction. Engelmann disease (progressive diaphyseal dysplasia), Down’s syndrome and even Turner’s syndrome^4^ have also been described.

The differential diagnosis includes neoplasia originating from bone or soft tissue. It is therefore imperative to correlate the laboratory and radiological findings with the clinical assessment. Other conditions that can cause localized soft tissue calcification include heterotopic ossification (myositis ossificans), calcified haemangioma or lymphatic malformation, teratoma, parosteal osteosarcoma and soft-tissue sarcomas, chronic renal failure, calcinosis universalis, hypervitaminosis D, milk-alkali syndrome and calcinosis circumscripta.^5^

The classical pathological findings at surgery include a "chalky" fluid extruded from pseudocapsules.^6^ Surgical excision is typically curative in children, but not usually in adults.

Martinez et al^7^ published an early paper outlining the radiological features of tumoral calcinosis, with more recent case reports having similar findings. On plain radiography, an amorphous, cystic, multilobulated para-articular mass, commonly on the extensor surfaces, is often demonstrated.^7,8^ Another consistent finding is a dense calcified mass that is homogeneous except for a "chicken wire" pattern of lucencies, which correlate histologically with fibrous septa.^9^ A fluid–fluid level correlating with cystic components of the mass can also be seen. CT findings are similar to that of plain radiography, but with more clarity to the lesion. Cystic components may show a layer of calcium within them, which is known as the "sedimentation sign".^7,8^ Despite the heavy calcific component, MRI signal can be variable. Zvaigzne et al^6^ discussed their radiological findings in a 16-month-old female with a mass along the scapular border. The MRI findings showed that the soft-tissue component was isointense to skeletal muscle on *T*_1_ weighted imaging, but heterogeneously hyperintense on *T*_2_ weighted imaging with multiple hypointense areas correlating with areas of calcium and fluid–fluid levels. Post contrast, the mass heterogeneously enhanced.^6^ In general, the lesions tend to be either (a) diffuse low *T*_1_ and *T*_2_ signal or (b) mixed high signal *T*_2_ with signal voids and low *T*_1_.^8^

Bone scintigraphy has shown avid uptake of tracer in some of these lesions in adults.^7^ Guveli et al^2^ describe in their case report heterogeneous uptake of ^99m^Tc tracer into tumoral calcinosis in the lower limbs of a 28-year-old female. It is well recognized that tumoral calcinosis behaves differently in adults and is more likely to recur following excision, as in the case described.^2^ There are, however, only a few cases in the literature that describe such findings in the paediatric population.^10^

## Conclusion

Our case describes the radiographic, MRI and bone scintigraphy findings of an unusual case of tumoral calcinosis in a child. Despite the rarity of this condition, it should be considered when encountering a calcified soft-tissue mass in all paediatric age groups. Its radiographic features have been described here and can aid in making a confident diagnosis.

## Learning points

1. Idiopathic tumoral calcinosis is a rare condition in children, which most commonly affects large joints, typically the hips, pelvis, elbows and shoulders.
2. On plain radiography, the typical findings are those of a cystic, multilobulated, para-articular calcified mass.
3. On MRI, lesions tend to be either diffuse low *T*_1_ and *T*_2_ signal or mixed high signal *T*_2_ with signal voids and low *T*_1_.
4. On bone scintigraphy, we have demonstrated avid tracer uptake.

## References

[1] Inclan A, Leon P, Camejo MG. JTumoral calcinosis. JAMA 1943; J121: 490–5.

[2] Guveli TK, Mulazimoglu M, Tamam MO, Tamam C, Tatoglu T, Ozpacaci T. JTc-MDP bone scintigraphy in a case with sporodical tumoral calcinosis. Indian J Nucl Med 2010; 25: 27–8. 20844668 10.4103/0972-3919.63598 PMC2934596

[3] Smack D, Norton SA, Fitzpatrick JE. Proposal for a pathogenesis-based classification of tumoral calcinosis. Int J Dermatol 1996; 35: 265–71. 8786184 10.1111/j.1365-4362.1996.tb02999.x

[4] Mukund A, Rawat L, Kumar A, Sharma GL. J Tumoral calcinosis in child Jhaving turner syndrome - a case report. Indian J Radiol Imaging 2006; 16: 349–52.

[5] Gan KB. Tumoral calcinosis: a case report and review of the literature. Br J Plast Surg 1982; 35: 177–80. 7082893 10.1016/0007-1226(82)90159-x

[6] Zvaigzne CG, Patton DJ, Kaur H, Trevenen CL, Kaura D. Subscapular tumoral calcinosis in a toddler: case report. J Radiol Case Rep 2012; 6: 10–17. 10.3941/jrcr.v6i6.967 PMC3558015 23378877

[7] Martinez S, Vogler JB, Harrelson JM, Lyles KW. Imaging of tumoral calcinosis: new observations. Radiology 1990; 174: 215–22. 2294551 10.1148/radiology.174.1.2294551

[8] Olsen KM, Chew FS. Tumoral calcinosis: pearls, polemics, and alternative possibilities. Radiographics 2006; 26: 871–85. 16702460 10.1148/rg.263055099

[9] Steinbach LS, Johnston JO, Tepper EF, Honda GD, Martel W. J J Tumoral calcinosis: Jradiologic-pathologic correlation. Skeletal Radiol 1995; 24: 573–8. 8614855 10.1007/BF00204854

[10] Leung YY, Lai R. Tumoral calcinosis: a case report. J Orthop Surg (Hong Kong) 2011; 19: 108–12. 21519090 10.1177/230949901101900125

